# Mild Bleeders: Diagnosis is Elusive in Large Number of Patients

**DOI:** 10.4084/MJHID.2016.049

**Published:** 2016-10-17

**Authors:** Mrinalini Kotru, Deepti Mutereja, Abhishek Purohit, Seema Tyagi, Manoranjan Mahapatra, Renu Saxena, Hara Prasad Pati

**Affiliations:** Department of Hematology, All India Institute of Medical Sciences, Delhi, India

## Abstract

**Background:**

Bleeding is a common clinical presentation. Even patients with mild bleeding disorders are extensively investigated for ascertaining the cause. The present study was conducted in order to evaluate the extent of the possibility of diagnosis in mild bleeding disorders.

**Material and Methods:**

This was a prospective study of patients referred for work up of mild bleeding for a period of 13 months. A complete blood count, peripheral smear examination, Prothrombin time, Partial Thromboplastin time and Thrombin Time, Platelet Aggregometry test, tests for von Willebrand’s disease and Platelet Factor 3 availability were measured.

**Results:**

164 patients presented with mild bleeding, in 114 of the patients a single site of bleeding was present. Epistaxis was the most common presentation (39%). Cutaneous bleeding (petechiae and purpura) was the next common site. History of a major bleeding tendency in the family was present only in 11 patients. The investigations showed that VWD (17/164), followed by clotting disorders (CD) mainly mild hemophilia (15/164) were the most common diagnosable cause. There were also 4 cases of hypofibrinogenemia. The disorders of platelets (Platelet function defects/PFD) were the least common (9/164). Rest 123 (75%) patients could not be diagnosed on the basis of these investigations and were labeled as Bleeding disorders – Unclassified (BDC).

**Conclusion:**

n our study, 75% of the patients with mild bleeding remained undiagnosed even after extensive laboratory workup, thus raising a very pertinent question that is it necessary that all mild bleeders submit to a broad battery of investigations, as the diagnosis continues to be elusive despite extensive workup.

## Introduction

A significant number of patients with mild bleeding symptoms are referred to hematologists for diagnosis. Unlike most of the severe and moderate hemostatic disorders which are diagnosable at in any tertiary care center, diagnosis of mild bleeders is not easy. It poses a challenge in diagnosis. In spite of the mild nature of the symptoms not needing clinical intervention most of the times, there is a pressing need for diagnosis because of the psycho-social impact of the bleeding on the patient. Moreover, patient self-assessment as well that of the physician of between normal variation and illness is subjective most of the time.

Mild bleeders do require extensive hematological workup for ascertaining the underlying cause. Screening laboratory defects have a limited predictive value and poorly relate to the clinical bleeding.^1^ In mild bleeding defects, laboratory tests may be normal, show subtle alterations or may be overtly abnormal. This study was done to ascertain the possibility of extending a precise diagnosis to mild bleeding disorders in a resource poor setting.

## Material and Methods

This was a crossectional, observational study conducted for a period of 13 months. All the patients were clinically evaluated for bleeding on a complete hospital form, which is used for routine clinical practice to maintain uniformity. All the patients with mild bleeding who were referred to our laboratory for work up were included in the study. Mild bleeding was defined as bleeding from any site which did not require any clinical intervention. All the cases with evident local cause of bleeding or on anti-inflammatory/anti- platelet drugs/antibiotics or who had more than three episodes of bleeding or showed leucopenia and/or thrombocytopena were excluded from the study (to eliminate the confounding effect of moderate to severe bleeders and so maintaining the focus on the mild bleeders only)

A complete blood count (to exclude thrombocytopenia and other cytopenic disorders) was made on Sysmex 1800i. Peripheral smear stained by Jenner-Giemsa was studied for platelet morphology. Prothrombin time (PT), Partial Thromboplastin time(PTT) and Thrombin Time(TT) were measured by using a clot-based assay (Diagnostica Stago) on automated coagulometer (STA Compact). Platelet Aggregometry test, tests for von Willebrand’s disease and Platelet Factor 3 (PF3) availability (Total and with ADP) were also performed.

### PF3 availability test

PF3 availability was measured with the Russel viper venom time (RVVT) test on platelet-rich plasma (PRP) with platelet count adjusted to 250 to 300×10^9^/L. The patient’s PRP was incubated with adenosine diphosphate (1mol/mL) for 0 and 20 min, and the clotting time was measured after the addition of 20 μmol/L of Russel viper venom. The samples were run in triplicate and the average clotting time was taken. An age and gender matched control-group was simultaneously run. The RVVT value for a standard PF3 availability ranged from 16.0 to 20.0 s at 20 min and from 24 to 28 s for 0 min (measured in individuals of either gender). Isolated PF3 availability defects were considered when PF3 availability was more than 19 s. with a normal PT and APTT and no other cause found.

### von Willebrand factor assay

Ristocetin induced platelet aggregation (RIPA) has been done for all patients who had prolonged APTT and low level of FVIIIc activity. VWF:Ag (VWF:Ag level assay) and VWF ristocetin co-factor assay (VWF:RCo), the most accurate tests for VWD, have been done. Patients with VWF:Ag and VWF:RCo below 50% and 60% respectively, were considered diagnostic of VWD. VWD patients were classified by VWF:RCo/VWF:Ag ratio. The patients having a ratio < 0.7 were classified as type-2 VWD and patients with ≥ 0.7 were considered as affected by type-1 VWD. If both VWF:Ag and VWF:RCo were less than 10% then the patients were classified under type-3 VWD (a severe type). Von Willebrand factor level was measured by using the Enzyme linked immunosorbant assay, ELISA kit. The average VWF Ag level was taken as 50% to 150%.

### Platelet Aggregation Studies

Platelet aggregation studies with agonists such as adenosine diphosphate (1.25 and 2.5 μmol/L), arachidonic acid (300 μmol/L), adrenaline (20 μmol/L), collagen (1.9 mg/L), and ristocetin (2 mg/mL) were performed using a chronolog platelet aggregometer in all patients (Chrono-log, Havertown, PA). PRP was used for aggregation studies. Bernard–Soulier Syndrome was considered when platelet aggregation with ristocetin was reduced and did not correct on the addition of normal pooled plasma. Glanzmann’s thrombasthenia was diagnosed when platelet aggregation with all agonists was absent except ristocetin. Arachidonic acid pathway defect was considered when aggregation with all agonists was reduced (was least with arachidonic acid) and dense granules were present. Storage pool deficiency was taken into consideration when platelet aggregation was reduced with all agonists, and there was absence or less than 15 granules/platelet found in the number of dense granules when counted under an electron microscope (Normal >15 granules/platelet). Unclassified was considered if ADP or ADR induced reduced or absent aggregation which, otherwise, was standard with the rest of the agonists. Ristocetin co-factor assays for von Willebrand factor have been done by using patients’ platelet poor plasma (PPP), normal PRP and ristocetin (30 mg/mL). The average value for VWF:RCo was taken between 60% and 150%.

## Results

During 13 months, 164 patients presented with mild bleeding. Their age ranged from 4 months a year to 78 years, median age being 17.5 years. There were 98 males and 66 females. Table 1 shows the clinical presentation of these patients along with the diagnoses. Bleeding from a single site was seen in 114 patients. Multiple sites bleeding were present in the remaining 50. Epistaxis was the most common presentation (39%). Cutaneous bleeding in the form of petechiae and purpura was the next common sites of bleeding. They were also the most common sites when patients had bleeding from multiple sites. History of conjunctival bleeding and umbilical stump bleeding was the least common. One case each of VWD and hypofibrinogenemia presented with umbilical stump bleeding. These patients had other bleeding sites as well (Table 1). The information about the duration of illness among 60 patients showed 38 patients were symptomatic for more than one year. 21 patients had symptoms for less than one year, and of them, 11 had symptoms for less than 6 months. One 48 year old patient became symptomatic with increased bleeding post operatively for the first time, and no cause was found even after extensive work up.

History of a significant bleeding tendency in the family was present only in 11 patients. Of these, VWD was diagnosed in 3 cases and Hemophilia A in 3. The remaining bulk did not report a positive family history.

The investigations made for diagnosis showed that VWD was the commonest cause of bleeding (17/164) disorder to be diagnosed, followed by clotting disorders (CD) mainly mild hemophilia (15/164). There were also 4 cases of hypofibrinogenemia. 123 patients (75%) could not be diagnosed by these investigations.

**Figure 1:**
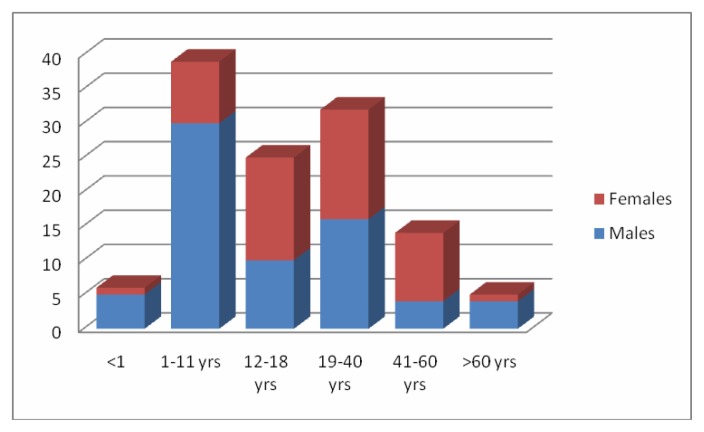
Distribution of the various causes of mild bleeding (in %) in males and females.

Figure 1 shows a slight female prevalence in the occurrence of VWD. The disorders of platelets (Platelet function defects/PFD) were the least common (9/164).There were one case of Bernard Soulier’s Syndrome, 5 cases of isolated PF3 availability defects and 3 cases of platelet function defects-Unclassifiable.

In the majority (75%) of cases, the diagnosis was not possible after the panel investigations for the same. This group was named Bleeding disorders – Unclassified (BDC). Umbilical stump bleeding, post-trauma and post-injection site bleeding were never seen alone without other bleeding sites. However, a definitive diagnosis was made in 66.7 & 70% of the patients who presented with these symptoms. On the other hand, the diagnosis was not made in the majority of cases who presented with epistaxis, cutaneous bleeding or menorrhagia.

**Figure 2:**
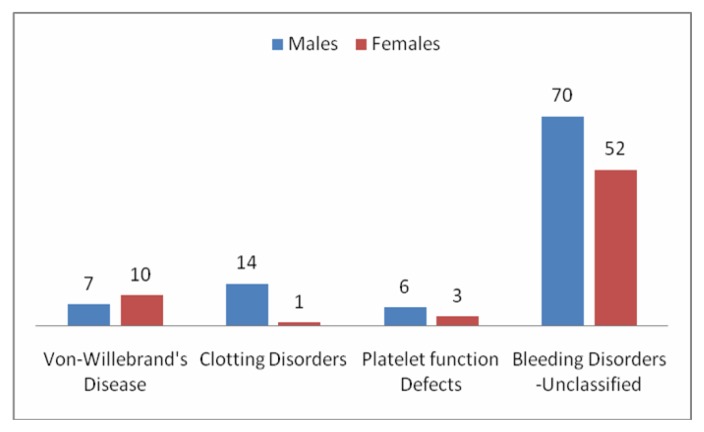
Age and sex distribution of patients with Bleeding Disorders-Unclassified.

Figure 2 shows the age and the sex distribution of this group. The age ranged from 1–60 years similar to the general trend. However, there was a slight female preponderance in the age group of 12–18 years and also between 42–60 years. 8 out of the 15 patients in the age group of 12–18years presented with menorrhagia as the only symptom. One patient had bleeding from multiple sites apart from having menorrhagia. In contrast, none of the females in the age group of 41–60 yrs. had gynecological presentations.

## Discussion

The diagnosis of mild bleeding disorders has been challenging. Distinguishing mild bleeding from physiological variations is clinically challenging. Many symptoms like epistaxis, gum bleeding, menorrhagia and petechiae have a very light discriminatory value and commonly occur in general population. However, others like umbilical stump bleeding and bleeding from eye and ear are highly suggestive of an underlying bleeding disorder. To confuse the matter further, disorders like VWD may be asymptomatic for most of their life and present for the first time in adulthood.^2^ Moreover, clotting disorders like hemophilia may occasionally have onset as mucocutaneous bleeds. Hence, relying on clinical parameters alone may become highly misleading.^3^ To address this issue and to maintain standardization in clinical assessment, many bleeding scores have been developed.^4^,^5^,^6^ Bleeding scores in specific disorders have also been studied for their clinical usefulness and discriminant power.^4^,^7^ However, despite the exhaustive list the confusion in classifying the patient as healthy or mild bleeder continues in most of the circumstances.

Dilemma increases further when the questions are put to the laboratory. A battery of investigations is required to investigate such cases, often with dismal results. In our group of mild bleeders, only 25% of cases could be classified successfully. Most other authors have also reported similar findings. Quiroga et al. in their study of 280 patients of mild bleeders also could classify only 113 patients. 59.6% of cases were put into a waste basket category of bleeding of unknown cause.^8^ They concluded that most patients had an unknown disease. Our experience with these cases of mild bleeding tendency also suggests that there is an extensive repertoire of causes leading to increased bleeding tendency which cannot be identified by routinely used tests to detect disorders of primary and secondary hemostasis.

Clinical presentation is also not discriminatory. Epistaxis is the commonest clinical presentation seen in our group of patients, followed by gum bleeds. A recent study from South Asian region which studied the spectrum of VWD also noted that easy bruising and epistaxis to be the commonest presentations in VWD patients.^9^,^10^ Both these clinical conditions have several local causes of bleeding which may not always be diagnosed. Gingivitis may both bleed as well as recur, mimicking a mild inherited bleeding disorder. Similarly, easy bruisability can also be a result of connective tissue disorders and variation in skin texture resulting spontaneous rupture of blood vessels. Menorrhagia, another presentation of mild bleeding disorders, more commonly has non hematological causes. In our study, 9 of the 15 cases of women in the pubertal age group of 12–18 years had menorrhagia as the initial presentation. Only one patient also had a history of bleed from other sites. Puberty menorrhagia can be a normal physiological variation. However, 4 out of these 9 patients was finally diagnosed as VWD. This brings out the fact that there is a thin line separating physiological from pathological bleeding which may result in over-investigating of some patients with above mentioned clinical presentations. A long follow up is essential to understand their clinical behavior and possible pathogenesis of such patients. Moreover, epistaxis, cutaneous bleeding, and menorrhagia have little significance when presenting in isolation as the majority of patients could not be diagnosed even after extensive evaluation. However, bleeding from more sites, especially when including the umbilical stump, had higher chances of being labelled with a specific diagnosis.

The list of investigations for bleeding disorders is quite exhaustive. However, it is relatively easy to diagnose diseases like severe hemophilia, type 3 VWD and well characterized platelet function defects like Bernard Soulier’s Syndrome & Glanzmann’s Thrombasthenia. However, it becomes difficult to classify milder forms of Hemophilia, rarer clotting defects and other variants of VWD with these first line tests. Moreover, the more unusual platelet function defects, rare disorders of the fibrinolytic system and coagulation pathway present as a major diagnostic challenge as they require more sophisticated assays and tests which are not available in all laboratories. Hence, they remain undiagnosed and unidentified most of the time. Tests for the primary hemostasis are limited and are of low sensitivity and specificity. Bleeding time is also going into disrepute due to lack of reproducibility and being invasive.^11^ Platelet aggregation tests also are subject to a lot of pre-analytical variables. However, a global platelet function test should be able to rule out the disorders of platelet function. In fact, Quiroga et al.^1^ suggest that mild bleeders with standard initial platelet aggregation tests should perform repeated work-up testing for clotting defects and VWD mainly. This is chiefly because VWD, Factor VIII, and some other clotting factors are also acute phase reactants and may be normal during initial testing. Then, repeat testing after a gap would be of value in picking some more cases.^1^

As others authors have also stated, the diagnostic efficacy of global tests for detection of coagulation and platelet disorders is low. 47% to 69% patients will remain undiagnosed even after extensive ranging laboratory investigations.^1^ The diagnostic efficacy in our study has been even lower (25.6%), probably because the patients included in the survey were investigated once. The data on subsequent re-evaluation has not been studied. Otherwise, of the patients who were diagnosed on first evaluation, most were cases of VWD. This is reflective of the fact that VWD is the commonest mild bleeding disorder. There was also a fair number of cases of Hemophilia precluding the general notion that Hemophilia does not primarily present as mucocutaneous bleeds.

## Conclusion

Diagnosis of mild bleeders is a challenge. Answers are not possible always, or rather mostly not possible with the commonly available battery of tests available. Even with an extensive panel of examens remains a huge gamut of yet unidentified cases which need more sophisticated investigations. However, more studies are required to identify the clinical signs and symptoms for a more judicious selection of patients to submit to extensive laboratory workup.

## Figures and Tables

**Table 1:** Distribution of the sites of bleeding and their causes.

Sites of bleeding	Total No.	Isolated Bleeding					Bleeding from multiple sites				
		VWD	C D	PFD	BDC	Total	VWD	C D	PFD	BDC	Total
Epistaxis	64	7	0	1	23	31	1	6	2	24	33
Cutaneous	56	3	4	2	18	27	1	4	3	21	29
Post trauma/injection site	23	0	0	0	13	13	0	6	1	3	10
Menorrhagia	24	3	0	1	16	19	2	0	0	3	5
Gums/oral cavity	18	0	1	0	9	10	1	1	1	5	8
Post sungica|/PPH	14	2	1	1	5	9	0	2	1	2	5
GIT,GUT	9	1	0	0	2	3	1	1	0	4	6
Conjuntiva	2	0	0	0	2	2	0	0	0	0	0
Umblical stump	3	0	0	0	0	0	1	1	0	1	3

VWD: Von-Willebrand’s Disease, CD: Clotting Disorders, PFD: Platelet Function Defect, BDC: Bleeding disorders-Unclassified, PPH: Post- Partum Hemorrhage, GIT- Gastrointestinal Tract, and GUT: Genitourinary Tract.
